# Understanding Family Risk and Protective Factors That Shape Child Development

**DOI:** 10.3390/children9091344

**Published:** 2022-09-02

**Authors:** Susan Yoon

**Affiliations:** College of Social Work, The Ohio State University, Columbus, OH 43064, USA; yoon.538@osu.edu

Understanding the various family characteristics and contextual factors that shape children’s health and developmental outcomes is important for promoting optimal child development. Research has suggested that family can have a salient influence on child development across social, emotional, physical, and cognitive domains. Despite a large body of existing studies on family environment and child development, much remains to be learned. Prior research has faced multiple conceptual and methodological challenges, including a reliance on mother-reported data (versus fathers) when examining parenting or other parent-related constructs. A lack of rigorous longitudinal data and conceptual complexity, such as changes in family structure over time, also adds to challenges. Furthermore, it remains unclear how risk and protective factors within families may contribute to child development among different subgroups of children and families across cultures. The articles presented in this Special Issue aim to overcome some of these limitations and advance the field’s understanding of the complex roles played by family risk and protective factors in explaining diverse developmental outcomes among children and youths.

This Special Issue features 18 articles that examine family risk and resilience among children and adolescents across developmental stages, ranging from early childhood to late adolescence/young adulthood. A wide range of child outcomes are examined in these studies, including children’s use of electronic devices [1,2], maltreatment experiences [3], mental health [4,5,6,7], school readiness and academic functioning [8], suicidal thoughts and behaviors [9], socioemotional development [10,11,12,13], and resilient/adaptive functioning [14,15,16].

Several salient family risk factors are identified and discussed in these studies. In Husa et al.’s study, pre-birth household challenges (e.g., homelessness, incarceration, substance use, intimate partner violence) were associated with lower reading proficiency and greater chronic absenteeism; these findings demonstrate the long-term negative effects of family risks on later child outcomes [8]. Similarly, Maguire-Jack et al. found that economic hardship, maternal substance use, intimate partner violence (IPV), and exposure to community violence were related to increased child abuse risk across three stages of child development: early childhood (age 3 years), young school age (age 5 years), and middle childhood (age 9 years) [3]. Furthermore, Showalter et al.’s qualitative study suggested that maternal IPV and IPV-related workplace disruptions threaten the safety and well-being of children [17].

Focusing on child physical abuse as a risk factor, Favre et al. identified distinct profiles of peer status among adolescents with and without physical abuse experiences. They found that higher levels of dissociation predicted membership in the rejected–unpopular group for adolescents with physical abuse experiences [13]. Interestingly, many unique family risk factors were found in studies that focused on problematic electronic use by children. Examining mobile device use among young children in Malaysia, Abdullah et al. found that when parents gave mobile devices to their children to make them sit still, children were more likely to become problematic users [2]. In Lee et al.’s study, the parent’s positive attitude toward media use and material rewards predicted the child’s daytime and nighttime media use, respectively, among children between 4 and 6 years of age [1].

Looking beyond risk factors, several papers focused on family strengths and protective factors related to childhood resilience. For example, Kassis et al. examined hedonic and eudaimonic well-being among adolescents with physical abuse experiences and identified distinct violence-resilient patterns and trajectories [14]. Not surprisingly, many studies found parenting or other parent-related constructs (e.g., parental relationships, parental support) to be key family protective factors in relation to positive child outcomes. In Quinn et al.’s study, positive parenting, operationalized as parents’ supportive verbal behaviors, was identified as a promotive factor for suicidal thoughts and behaviors in a national sample of justice-involved Black youth aged 12–17 [9]. Zhan et al. examined associations among emotion regulation, parental relationships, and psychotic-like experiences among adolescents (mean age 17.9 years) and found that positive parental relationships buffered the adverse effects of maladaptive emotional regulation patterns on distress from psychotic-like experiences [7]. Focusing on Black youth affected by community violence, Donte et al. found that positive parent relationships and parent bonding predicted resilience to adverse community experiences [4]. Barnhart et al. found that family resilience (e.g., staying hopeful, drawing on strengths, working together when facing a problem) was positively associated with higher levels of child and adolescent flourishing [15].

Compared with the many papers that have examined psychological and relational strengths as family protective factors, fewer studies have considered material resources, such as food, housing, and financial security, as potential protective factors. Kobulsky et al. found that food security and housing stability buffered the negative effects of abuse and neglect on adolescent adaptive functioning [16]. In line with Kobulsky et al.’s study—but focusing on low-income Hispanic families and their young children during the COVID-19 pandemic—Cabrera et al. found that positivity (e.g., staying optimistic about the future) and economic support (e.g., WIC/SNAP) buffered the adverse effects of economic risk and helped parents to manage their parenting stress and stay engaged with their children [11]. Notably, Evans et al. found that having family support and material support predicted greater life satisfaction among youths with a history of out-of-home care; highlighting the importance of both relational and material resources as important family protective factors [5].

It is important to highlight the papers in this Special Issue that focused on fathers as a source of protection and resilience. Yoon et al. examined the role of father involvement in the development of social, behavioral, and cognitive functioning among low-income children (age 5 years and under). Cognitive stimulation by fathers was found to be an important promotive factor for positive child socioemotional and cognitive development [10]. In Donte et al.’s study, father bonding was associated with a reduction in pre-exposure prophylaxis (PrEP) stigma among young Black and Latino men (aged 16–24 years) [18]. Olofson and Schoppe-Sullivan used a newly developed coding system for measuring parenting behaviors and reported that fathers’ and mothers’ behaviors were differently associated with children’s social–emotional development. Fathers’ allowance of greater autonomy and lower overprotection predicted lower levels of internalizing symptoms; at the same time, when mothers challenged children’s regulatory competence, lower levels of externalizing symptoms and higher levels of competence were predicted among toddlers [12].

Notably, this Special Issue includes studies that represent diverse regions, cultures, and contexts. The international studies featured in this Special Issue involve study participants from South Africa and Canada [6], China [7], Switzerland [13,14], Malaysia [2], and South Korea [1]. Findings from these studies offer valuable insights that enrich our understanding of cultural differences and nuances related to the influence of family risk and protective factors on child development. Cameranesi et al. drew from the multisystemic resilience framework to examine positive adaptation following exposure to family adversity using two different samples: Canadian adolescents and South African adolescents. They found different results between the two samples, with peer support serving as a protective factor against family adversity for Canadian adolescents but not for South African adolescents. Interestingly, a strong appreciation for community traditions was positively and significantly associated with conduct difficulties for South African adolescents. See Cameranesi et al. [6] for further discussion of these novel findings.

Together, the collection of articles featured in this Special Issue validate the important role of family in determining child outcomes; further contributing to our understanding of the various ways in which family risk and protective factors may promote or inhibit positive child development. All the works included in the Special Issue provide invaluable contributions to the field of family science and child development. The included works also add support to the need for continued investigation and rigorous research to disentangle complex relations among family risk and resilience factors and child outcomes.

## Data Availability

Not applicable.
